# Roles of technologies for future teaching in a pandemic: activity, agency, and humans-with-media

**DOI:** 10.1007/s11858-022-01429-4

**Published:** 2022-09-06

**Authors:** Jhony Alexander Villa-Ochoa, Juan Fernando Molina-Toro, Marcelo C. Borba

**Affiliations:** 1 Universidad de Antioquia, Medellín, Colombia; 2 São Paulo State University (UNESP), Rio Claro, SP, Brazil

**Keywords:** COVID-19, Technology, Agency, Activity system, Humans-with-media

## Abstract

Research literature on the role of mathematics teachers during the COVID-19 crisis shows that teacher preparation for emergency situations is required. In reporting on this exploratory study, we present and analyse lesson plans created by seven future teachers for mathematics classes during the pandemic. Data were collected between April and October 2021 from 16 four-hour class sessions in a Mathematics Degree Program at a public university in Medellín, Colombia. The notion of Humans-with-Media and the Learning by Expanding theory were used as frameworks to understand what roles prospective mathematics teacher (PMTs) assign to technologies for teaching in pandemic conditions. The PMTs’ uses of technology for teaching mathematics during a pandemic were categorized. The results show that technology was used to reorganize and reproduce mathematics teaching practices. This report addresses the impact of technology on the activity system, and we conclude with a discussion of opportunities and limitations of students’ conceptions about teaching and technology during a pandemic.

## Introduction

Since 2020, the spread of COVID-19 has caused crises throughout most of the world. Where feasible, schools and universities adopted digital technologies, such as Internet-connected computers, laptops, and mobile phones to provide continuity in education while in-person learning was not possible. Research on the impact of COVID-19 in education has grown significantly worldwide. Mathematics Education researchers have investigated subjects such as required competencies and curriculum development in local contexts (Maraví Zavaleta, 2021; Aguilar & Castaneda, 2021), perceptions, uses and barriers in educational environments using technologies (Yılmaz et al., 2021), and family interaction with schools regarding children’s mathematics education (Leon, 2021). Such studies have documented the impact of the pandemic on education in specific communities and authors have discussed its implications in terms of projected strategies and recommended guidelines. The move towards remote education has renewed old discussions on education in times of emergency (INEE, 2010; Parra-Zapata & Villa-Ochoa 2022), generating useful response guidelines and research topics to be developed during the current health emergency in order to understand education in the long term, since these changes are expected to produce permanent transformations (Parra-Zapata & Villa-Ochoa, 2022).

Teachers, as members of one of the main groups of educational actors, have had to change their routines and, at the same time, find ways to give continuity to teaching processes and to meet students’ learning goals and expectations. Teachers’ proficiency in teaching during emergency situations that require remote teaching is another important subject in recent research. A survey of secondary school teachers from 20 countries examined the proficiency of teachers and institutions in emergency online teaching (Howard et al., 2021). The results of the study showed four perception profiles, namely, high, medium, low, and mixed perceptions of proficiency to teach online; each profile is associated with the professional and institutional support that is needed. In another survey with German early career teachers, König et al., (2020) found that, during the first phase of lockdown, most teachers reported that they maintained communication with students and their parents; the study showed that information and communication technology (ICT) tools and teacher’s digital competence and related preparation opportunities are critical for adequate adaptation to online teaching during COVID-19 school closures.

In over exploited countries, teachers had additional challenges; besides their own preparation needs, they had to provide teaching continuity in conditions of poor access to technological resources and limited Internet access. In Libya, Palestine, and Afghanistan, Khlaif et al. (2021) showed how some teachers found creative solutions to engage students in learning and minimize educational disruptions. The study revealed how teachers developed technological skills and designed appropriate digital content: teachers, together with the local community, created community centers for students from poor families. In Latin America, Borba (2021) reported that, despite students’ connectivity difficulties, and while the social reality of teachers in the region entails limited economic resources to access certain technologies and little time to spend on their education, teachers came up with creative alternative ways to continue teaching. In Latin America, some experiences involving methodologies that combine projects and digital videos contributed to the preparation of mathematics teachers (Vargas-Díaz, 2021). Other studies revealed the need to prepare teachers to design learning environments that promote better class interaction, avoiding situations in which they merely continue their traditional classes even while using electronic devices (Calle et al., 2021).

The research presented so far shows that, although the current general pandemic imposed favorable conditions for technology integration, some important problems still persist; in addition, the pandemic has generated new learning needs, such as, in conditions in which teachers have little time, excess of information and restricted access to appropriate devices (Borba, 2021; Engelbrecht et al., 2020). Considering these needs, in this paper we analyse a course for future teachers focused on technology integration in mathematics teaching. The course was reorganized during the pandemic, so that beyond promoting technology integration, it also showed how to design and implement necessary reorganizations to promote the learning of mathematics in crisis conditions. Specifically, in the present study we examined relevant phenomena and challenges focused on prospective mathematics teachers’ (PMTs’) learning. Some studies have highlighted that remote emergency education has fostered favorable conditions for the integration of technologies (Borba, 2021; Naidoo, 2020). However, given the uncertainty and continuous changes in school attendance, the new conditions demand flexible designs adapted to varying institutional contexts. To build on these studies, in this paper we focus on a different research question: What roles do PMTs assign to technologies for future teaching in pandemic conditions? First of all, we discuss the notions of Expansive Learning and Humans-with-Media (H-w-M); then we present our methodology, results, discussion, and conclusions.

## Notion of learning from an expansive perspective

Expansive Learning theory was formulated with the purpose of guiding the transformation of collectives of people in organizations and workplaces (Engeström et al., 2013). It appeared as an alternative to learning theories that focused attention on human cognition (Engeström, 1987), and constituted the foundation for analyzing artifact-mediated actions performed by groups of subjects. In this theory, learning goes beyond the acquisition of disciplinary knowledge at an individual or collective level and involves the creativity of the subjects who, by joining forces and presenting their points of view, create something new and build new experiences around something that did not exist previously (Sannino et al., 2016). The theory offers different ways to analyze, interpret and recognize actions carried out by groups of people sharing the same activity, and allows the researcher to understand how social and cultural transformations are constituted in the environment in which the activity is developed.

**Fig. 1:**
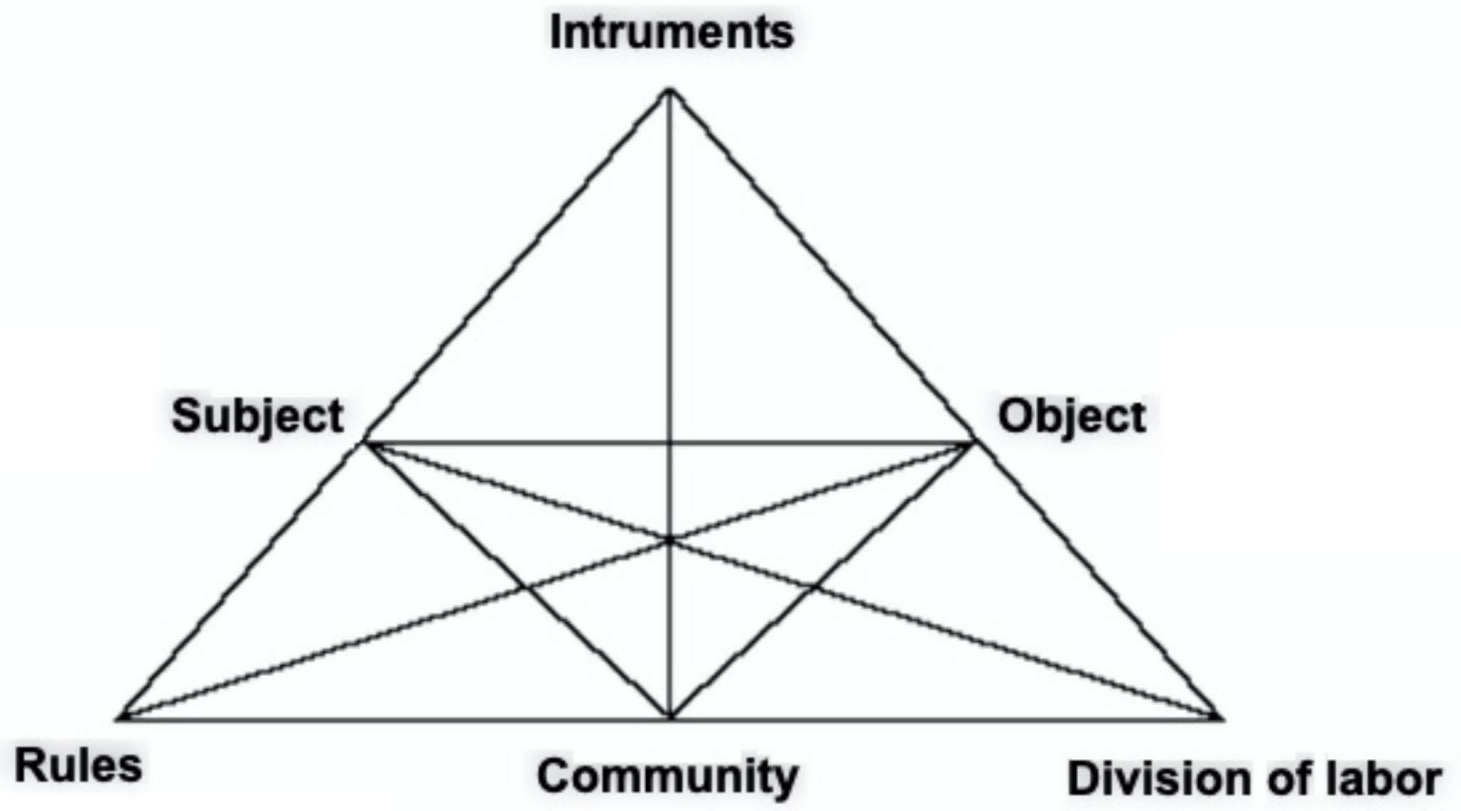
Engeström’s (1987, 2015) activity system structure

According to Engeström (2015), human learning is related to the various types of social activities established over time in society. These diverse social activities drive the learning activities, implying that human learning processes are also diverse and continually changing. To better understand how subjects learn, what factors promote and drive their learning, and what they can learn, the theory presents tools to identify knowledge expansion and qualitative transformations in activity systems. These systems (see Fig. 1) make it possible to identify the subjects that participate in an activity, their community, the instruments of mediation, the rules and roles assumed by each participant, and the object that directs the activity.

In the triangular model (Fig. 1), it is observed how human activity is reorganized, how its multiple voices are interrelated and how learning actions are formed. As part of the analysis of activity systems, the theory proposes to identify contradictions that arise in their development and to evaluate how new forms of activity arise in their solution. For Engeström & Sannino (2010), contradictions can appear at different stages of expansive learning in the form of conflicts, dilemmas and disturbances that can be identified in the discursive expressions of the subjects. In correspondence with the structure of activity systems, this implies analyzing not only what the subjects externalize but also the means and forms in which these expressions occur, and the object of the actual activity.

## Humans-with-media as an epistemological perspective

Borba & Villarreal (2005) pointed out that when humans interact with technologies, they reorganize their thinking according to the possibilities and constraints that such technologies offer. Borba (2012) added that technology reorganizes “other dimensions of human life. Knowledge is produced by collectives of humans-with-media not only through cognition but also as a result of affective and value-related motives” (p. 805). Based on Activity Theory, Borba & Villarreal (2005) pointed out that technologies condition human thought, but do not determine it. Thus, they avoid technological determinism and place humans as active subjects, capable of recognizing, adopting and modifying technology to meet significant objectives, according to its possibilities and limitations. Neither humans nor technologies are independent actors; on the contrary, they mold themselves reciprocally according to the environmental conditions and contexts in which they interact. In this perspective, technology, humans, knowledge, emotions, culture, environments, and other internal and external factors are key components of a H-w-M system. Such systems share the agency among humans and media or things. Things and media have ‘fuzzy agency’, impregnated as they are by humanity participating in goal-oriented activity, as stressed by Borba (2021):

The notion that both humans and non-humans have agency is part of an effort to model artifacts—in particular, pieces of software, hardware, and the Internet of Things (i.e., things that are connected to the Internet)—as the historical, social, and cultural factors in the collective that produces knowledge. It stresses a view that knowledge is produced (both from a philosophical and a psychological perspective) by humans-with-artifacts. (p. 391)

## Methodology

### The course and the participants

The research scenario was a course of technologies for mathematics teaching of a bachelor’s degree in teacher education. The course was compulsory for all students in the program and aimed to offer conceptual, theoretical, and didactic preparation for technology integration in mathematics teaching. The course was organized into the following four thematic units: (i) technology integration in mathematics classes, (ii) experimentation and technology in mathematics classes, (iii) study of mathematics supported by software, mobile devices, and video games and (iv) mathematics education, Internet, and augmented reality. The course was offered in the last two years of the bachelor’s program, and courses in mathematics (calculus, algebra, statistics, etc.), pedagogy (curriculum, policies, psychology, etc.) and didactics (number, geometry, algebra, and statistics) were prerequisites.

The course consisted of 64 h distributed over 16 weeks. During the pandemic, students attended synchronous sessions of three hours per week plus one hour of independent work; in addition, students had one extra hour for personalized advising with the course teacher (the second author of this paper). The first pandemic-specific version of the course, which ran from August to December 2020, considered reflections on lockdown conditions and on how technologies were supporting the continuity of teaching. The second version of the course ran from April to October 2021. The design of this version took into consideration ongoing discussion and reflection on what was happening in educational contexts during the pandemic, as well as reading and discussion of scholarly articles reporting actions and research findings on technology during the pandemic. Even when the course was redesigned to be remote during the emergency, Borba’s (2012) recommendations were considered, including the combination of synchronous problem-solving sessions with asynchronous individual interaction and interaction among the professors and the PMTs. The professor started all synchronous collective sessions, and most of the individual asynchronous interactions, by asking PMTs about their experiences in the pandemic and in other classes. Their experiences were used as a basis for discussion and collective learning.

The course also included multimedia presentations illustrating how classes and other academic processes were being reestablished in public and private schools through a process called “alternation” (alternancia in Spanish). The alternation represented a gradual return to the classroom, in groups of 15 students at most, and with a new distribution of work times and curricular areas. The course teacher, who also worked in a public school with students of ages from 15 to 18 years, shared information, and images of the alternation process at his school. The inclusion of this subject in the course was based on the need for PMTs to have first-hand experiences with available technologies, their means of operation, and the opportunities and limitations they can offer during remote teaching. As part of the assessment process, students developed a lesson plan (LP) that included the use of technology in mathematics teaching in the context of the current pandemic. Throughout the semester, oral presentations of the LP progress were required, and students also had the option of seeking advice from the teacher outside of class.

All classes and advice sessions took place using the Google Meet platform and the students consented to the recording. These recordings were made available to the students for later review, and to the teacher and researchers for analysis. Eight PMTs initiated the course, but only seven of them completed it. In total, five LPs were developed. The title of each plan is given in the Table 1.

**Table 1:** LPs developed by PMTs

No	PMT Pseudonyms	Title
CP_1	Paola, Alejandro, José	LP design using the software Homestyler
CP_2	Ana	LP with virtual tools
CP_3	Jorge	LP on triangles and their classification
CP_4	Isabel	LP on proper and improper fractions
CP_5	Luis	LP on length measurement

### The data

During the course, digital documents, videos, and observation notes were produced in each class session. Each file was encrypted and stored in the cloud. Each of the three researchers had access to the files. To answer the study’s research question, a subset of the data was extracted. The extracted data contained the following. (i) Nine videos corresponding to class sessions focused on technology issues during the pandemic. In these sessions, students spontaneously participated and presented their vision of education in the midst of a pandemic. (ii) Two videos of discussion sessions with students divided into two subgroups. In this space, each student had the opportunity to participate and answer questions related to their point of view about future changes in educational processes after the pandemic, and about the roles that students and teachers should assume in such processes. (iii) Two videos of oral presentations of the LPs, including the question-and-answer session where the presenters discussed their LP with the teacher and the other classmates. (iv) Five digital documents with the respective LPs.

### Data analysis

Data analysis consisted of an iterative process of back-and-forth between the concepts of expansive learning, H-w-M, and the data. Throughout this process, we followed the guidelines of thematic analysis. This type of analysis is “a method for identifying, analysing and reporting patterns (themes) within data” (Braun & Clarke, 2006, p. 79). Thematic analysis can be conducted through six phases, namely, Familiarisation, Coding, Theme Development, Reviewing, Defining Themes, and Producing the Report (Braun et al., 2019).

The researchers reviewed the collected data and wrote down their impressions on the relevance and sufficiency of the data to answer the research question. Subsequently, the data were introduced in the software Atlas.ti and a first analysis was carried out, seeking to identify fragments of data that would evince students’ learning. The coding was conducted attending to “an inductive orientation, where the researcher starts the analytic process from the data, working ‘bottom-up’ to identify meaning without importing ideas” (Braun et al., 2019, p. 853). Therefore, there were no pre-defined categories, and the analysis was done having in mind just the research question (Borba et al., 2018). The extracted fragments were analyzed and discussed among the researchers, considering the concepts of expansive learning and H-w-M. As a result of this discussion, a new coding system was constructed and a second deductive coding process was conducted “where the researcher approaches the data with various ideas, concepts, and theories, or even potential codes based on such, which are then explored and tagged within the dataset.“ (Braun et al., 2019, p. 853). In this coding, Atlas.ti was used.

Following this phase, the research team met for discussion. Given the pandemic conditions in which this study was carried out, H-w-M was used in order to account for the uses that PMTs made of technologies to continue teaching mathematics in the midst of a pandemic. Within this framework, the notion of ‘use’ transcends the ‘presence’ of a technology in the system and focuses on how this technology can offer possibilities to achieve the subject’s objectives and satisfy their explicit needs (agency). These technologies formed collectives with the PMTs and had agency. Based on this idea, technologies presented in the LPs were identified and evidence was sought to understand the following questions: What were they used for? What potential actions could they promote in the learners? How were they articulated with other technologies in the LP? What pandemic conditions were addressed? These questions were key to identifying possible agencies in the LPs (Borba, 2021; Kaptelinin & Nardi, 2006). The results of these analyses are presented in Sect. 5.1.

Subsequently, the data were reviewed again to determine how the course activity system was configured and to identify how pandemic conditions reorganized the components of the activity system. For this purpose, the Yamagata-Lynch (2010) criteria were used to analyze the activity system, and the Mwanza (2001) model was used to answer the following questions. What type of activity is carried out? Why is the activity carried out? (object). Who carries out the activity? (subjects). With what means is the activity carried out? (instruments). What rules regulate the development of the activity? (rules). In which environment does the activity take place? (community). Who is responsible for carrying out the activity? (Division of labor). Through this analysis, the components of the triangular model (Engeström, 1987) were identified. In addition, data fragments from the previous phase were reviewed and grouped into potential themes with their respective coding (PSI, Pandemic-subjects-instruments; PR, pandemic and rules; PC, pandemic-community; PD, pandemic and distribution of work). In a collective session of the researchers, themes that were able to tell a coherent and insightful story about the data in relation to the research questions were selected (Braun et al., 2019). Subsequently, the selected themes were reviewed again in order to avoid “analytic thinness” or “conceptual overlap” (Braun et al., 2019), paying attention to the “robustness” of the evidence that supported them, the clear definition of each theme, the concepts involved and the relations between those concepts. Finally, according to the analysis, four themes (See Sect. 5.2) were selected to explain how the pandemic conditions affected the nodes of the activity system proposed by Engestrom (1987).

## Results

### Roles of technology in the LPs

Based on H-w-M, the notion of ‘use’ was explored (Sect. 4.3) and the Class Environment, Purposes, Technologies, and Mathematical Goals were identified. This made it possible to identify functionalities, activities and how technologies are involved in LPs. Two roles that (PMTs) assign to technologies were recognized, namely, technology as a reorganizer of teaching during the pandemic, and technology that reproduces before-pandemic conventional teaching.

#### Technology as a reorganizer of teaching during the pandemic

In two LPs (those of CP_1 and CP_2), digital technology was employed in order to create opportunities to explore, experiment and foster some mathematical process. The LP of CP_1 was based on the promotion of visualization processes with Homestyler to observe three-dimensional objects from different points of view and to characterize a body in terms of two-dimensionality and three-dimensionality, while CP_2 proposed a flipped classroom structure to work on reasoning skills based on plane transformations. Both LPs were structured in several sessions for blending asynchronous and synchronous activities. In Table 2, some excerpts on the participation of technology in class environment, purposes, and mathematical goals in the two LPs are presented.

**Table 2:** LPs using technology to explore, experiment and foster mathematical processes

Participation of technology	CP_1	CP_2
**Class Environment**	**Features**: *“This lesson plan is dynamic and allows us to present it during a pandemic, because it can be applied simultaneously face-to-face and virtually with the students. " [Doc: CP_1]*” **Stages**: The synchronous session was structured in three stages, namely, (i) introduction and explanation by the teacher of the topic to be worked on, (ii) video presentation on the Homestyler tool, and (iii) activity to be developed by the students through the Homestyler application.	**Features**: *“The flipped class considers three moments: before, during, and after the class”. Additionally, she described other characteristics of this type of class: “(i) technology and learning activities are two key elements of this pedagogical model; (ii) Each class can include videos of 3 to 7 minutes of duration, which are projected on a weekly basis; the videos are recorded by the teachers and incorporate complementary educational resources such as books, podcasts, Internet sites, among others; (iii) the class should promote collaborative learning and critical thinking. In the inverted classroom there should be no frustration. It is essential that those who did not learn something have the support of their group”* [Ana, Doc: CP_2]. **Stages**: This lesson plan includes three stages. The first was named “home learning activities”; the second, “learning activities”, and the third, “evaluation moment”.
**Purposes**	“*Provides students with a classroom environment that allows them to interact with the technological tool Homestyler, letting them understand mathematical concepts, particularly the topic of visualization*” [Doc: CP_1].	“*Recognize the Cartesian plane as a two-dimensional system that allows to locate points as a graphic or geographical reference system*”; “*locate, describe and represent the position and trajectory of an object in the plane*” [Ana, Doc: CP_2].
**Technologies**	Videoconferencing platforms, Homestyler	Videos, videoconferencing platforms, educational platforms (e.g. Kan Academy), Internet, GeoGebra, paper and pencil
**Mathematical goals**	**Exploration and Conceptualization**: the software sought to *“Observe and represent three-dimensional objects […]”. “identify and describe properties that characterize a body in terms of two- and three-dimensionality and solve problems related to the composition and decomposition of shapes”*. **Procedural**: “*Apply transformations to figures in the plane […]*” *“Acquire skills through the use of the Homestyler tool to construct and model planes in 2 and 3 dimensions”.*	**Exploration and Procedural**: In GeoGebra (Fig. 2), the content of the activity is a triangle with vertices A, B, C (free points) and a slider controlling the translation vector magnitude. Students could manipulate the vertices A, B or C, and the slider; the triangle A’B’C’ represents the translated figure, according to the translation vector. **Complementarity and collaboration**: In the ‘home learning activities’ stage, students are invited to complete, using pencil and paper, a video quiz which offers explanations and questions about translations in the Cartesian plane. The second stage contains GeoGebra activities: some to be solved individually, others in a collaborative way. **Assessment**: Khan Academy videos and online assessment resources.

**Fig. 2:**
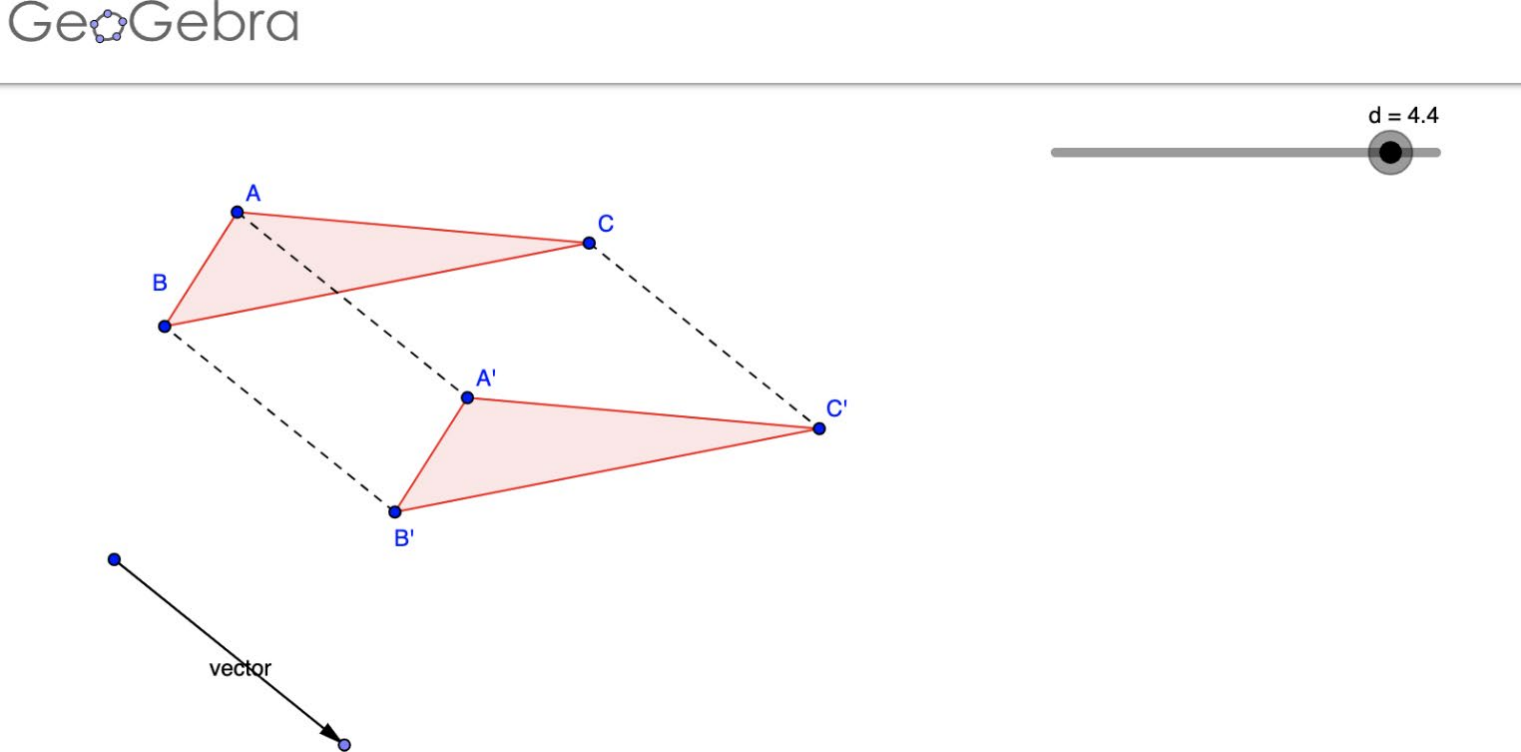
Example of task in GeoGebra

In Table 2, two types of technology can be identified. On the one hand, there are Internet-based technologies that enable contact between subjects, communication, access to information, etc. These technologies contribute to the creation of hybrid learning environments for participating, interacting, and producing mathematical knowledge. On the other hand, there are mathematical technologies (e.g., GeoGebra in Fig. 2), which the PMTs integrated with Internet-based technologies to foster mathematical knowledge. These aspects were decisive in characterizing the agency of PMTs and the agency assigned to technologies, as we discuss in Sect. 6 of this paper.

#### Technology that reproduces before-pandemic conventional teaching

The other LPs (CP_3, CP_4 and CP_5) were focused on teaching mathematical themes. CP_3 worked with triangles and their classification, using GeoGebra on mobile devices. CP_4 studied proper and improper fractions with a special emphasis on figural representations and making reference to everyday objects or situations. CP_5 intended for students to identify and use instruments to measure lengths and made use of educational videos from YouTube and GeoGebra, in which tasks are presented that invite them to establish measurements of figures from a given geometric pattern. In addition, with the use of Cartesian coordinates and points in the plane, exercises were proposed to establish measurement patterns with the app GeoGebra for mobile devices. Like those of CP_1 and CP_2, these LPs were organized in several sessions for blending asynchronous and synchronous activities.

**Table 3:** Use of technology that reproduces conventional teaching

Participation of technology	CP_3	CP_4	CP_5
**Class Environment**	**Features**: Due to the alternation “*only a maximum of 18 students per group is allowed, so it is recommended that the teacher make a presentation on any platform (It could be meet, Zoom, Teams or Edmodo) and, after making the presentation, share it by institutional email or WhatsApp groups, so that all students have access to the topics developed in class and can participate from their homes, without the need to separate the group into two parts*” [Jorge, Doc: CP_3]. **Stages**: Two sessions of 45 min each; the first one consisted of a review “to deepen students’ knowledge about polygons (triangles) and about the construction of geometric figures in the plane” [Jorge: Doc: CP_3]. The second session included an explanation of the use of GeoGebra for the study of geometry and an exploratory activity.	**Features**: *Structured sessions with guidelines to distribute time and tasks in class, a list of resources to be used in alternating conditions, a list of examples, exercises, and links to videos and GeoGebra Classroom activities, adapted to different session stage.* **Stages**: three two-hour sessions. The first session began with a set of questions about proper fractions, to assess students’ previous knowledge. The second session was focused on improper fractions and a video was proposed as a complement was a cartoon explaining improper fractions, with related examples and exercises. The third session was planned to answer students’ questions and to present the learning obtained using GeoGebra.	**Features**: Structured session with guidelines to distribute time and tasks in class. **Stages**: Four moments were identified. (i) started with a short Catholic religious’ activity. (ii) review the guidelines and agreements for online classes. (iii) check and review of students’ previous knowledge, then planned a session with paper and pencil activities. (iv) a practical session with the smartphone.
**Purposes**	“Review the classification of triangles, considering their properties and their role in the construction of plane figures (polygons), using the cell phone as a learning tool and the software GeoGebra as an interactional means” [Jorge, Doc: CP_3].	“Recognize and identify differences between proper and improper fractions as observed in the graphs” and “solve equations using fraction graphs as support” and “practice operations with fractions using everyday examples” [Isabel, Doc: CP_4].	to calculate and to search and represent information about geometric figures. [Luis, Doc: CP_5]
**Technologies**	videoconferencing platforms, GeoGebra, WhatsApp, email, TV.	Cartoon videos, videoconferencing platforms	GeoGebra through Smartphones, YouTube videos
**Mathematical goals**	**Definition Introducing**: Jorge offered conventional definitions of triangles and their classifications according to the length of their sides and the magnitude of their inner angles. He proposed a closing activity in which he presented four different triangles and asked the students to use ruler and protractor to measure sides and angles and classify the given triangles. **Procedural**: an activity to explore what happens when the position of the vertices of a polygon is changed and a set of exercises in GeoGebra Classroom at the end of the session.	**Conceptualization**: a video with a family story, where fractions are conceptualized and exemplified by splitting and sharing a pizza was proposed. To work with proper fractions, she suggested the presentation of examples from the student’s booklet: “the examples that this guide contains will be shown and explained with the purpose of having greater clarity of the subject. Cartoon videos were used to provide conventional content information (definitions, examples, exercises). One GeoGebra applet allowed the superposition of two figures representing fractions and offered a figural representation of the product of fractions. **verification**: Isabel suggests teachers “check through the GeoGebra application if the solved exercises are correct” [Doc: CP_4].	**Procedural**: Luis used GeoGebra as a tool for graphing and calculating measurement and surfaces and fostered knowledge of concepts and procedures for the calculation of areas of geometric figures.

These LPs included Internet-based technologies such as: cell phone, GeoGebra, videoconferencing platforms, instant messaging, and TV screen. On the one hand, GeoGebra was used to present a dynamic and exploratory version of the contents (CP_3, and CP_4) and for calculating and lengthening measurements (CP_5). Overall, the participation of technologies in the mathematics teaching environment reproduced traditional goals, practices, status quo of pre-pandemic classes. Like CP_1 and CP_2, videoconferencing sessions were used to deal with institutional pandemic conditions; however, their principal function was to reproduce information. As we discuss in the next section, it is also possible to recognize other forms of agency based on technologies.

### Pandemic conditions and reorganization of activity system nodes

#### Pandemic: subjects and instruments of activity

The analysis of the videos and the documents show the PMT’s interest in focusing their LPs on teaching mathematical themes. During the progress presentation session, the following excerpts were presented:

“*I want to tell you that I first chose [the subject of] solids of revolution because I already had an exercise to do with GeoGebra 3D.*“ [Alejandro, Video 08/13/2021].

“*Yesterday I logged-in to GeoGebra. I was interested because there are some related topics that I’m teaching at the school now.*“ [Ana, Video 08/13/2021].

As shown in Sect. 5.1, the other LPs focused on teaching about triangles, their properties and length measurement, presenting the set of technologies used for information, communication, conceptualization, visualization and training. In the Expansive Learning perspective (Engeström, 1999; Borba, 2021; Souto & Borba, 2016), both technologies and thematic content can be considered activity production tools. PMTs took into consideration the fact that pandemic-related social distancing changed the type of relationships, interactions, information access and communication dynamics between teachers and students; therefore, they proposed the use of technologies with specific roles to cope with these limitations (e.g., videoconferencing platforms, instant messaging, social networks). In some cases, the opportunities offered by technology in pandemic conditions determined the choice of thematic contents in the LP; for example, Alejandro said “*Professor, I* […] I *changed the exercise, so I do not have everything planned yet* […] *I was thinking of inscribing a triangle in a circle* […] *it can be done very well with GeoGebra on the cell phone*” [Video 13/08/2021]. It is noteworthy that, across the different LPs, sessions are organized into the same sequence, roughly speaking: presentation of content (definition and exploration), exemplification, exercises, and evaluation through exercises. In the LPs, technologies such as videos and repositories played an informative role, providing access to definitions, explanations, examples, and assessments. In the case of GeoGebra, the PMTs used pre-pandemic resources; these resources also played an informative role, but, additionally, sliders and figure movement were used to enhance students’ visual experience and exploration of regularities, definitions, and properties.

According to the PMTs, due to the pandemic, technology acquired the role of “learning object” within the activity system due, on the one hand, to the presence of contradictions between the nodes of the system, since the integration of new instruments to the activity was required. On the other hand, this role was due to the potential transformations of the object that led temporarily to focus attention on the study of new tools to incorporate into the work with students in times of pandemic. In this regard, the PMTs acknowledged its mandatory presence in school environments. For example, Jorge pointed out “*technology is here to stay*” [Video, 10/09/2021] and Isabel added: “*the challenge we are facing […] we have to assume virtuality* […] *we have to find the means to learn things about technology*” [Video 10/09/2021]. These excerpts suggest that, for the PMTs, technology has gained a character of ubiquity, and Internet-based technology has become indispensable for the continuity of mathematics education.

#### Pandemic and rules in the activity system

In the Expansive Learning Theory, the rules within an activity system allow it to take into account the norms followed by the subjects of the activity (Lektorsky, 2009). In the LPs, it was observed that the presence of the pandemic directly and indirectly affected the rules of the activity system. Three types of rules were observed that conditioned the activity of designing a mathematics class in pandemic conditions. Such changes illustrate the agency of the tools (Borba, 2021).

Firstly, *governmental and institutional dispositions* were followed; in the LPs, PMTs clearly took care to design the class for an alternate model; this model was decreed by the national government for public institutions in June of 2021. In particular, the case of Ana shows how the school conditions influenced her choice of the inverted model for her class design; likewise, Jorge repeatedly pointed out that “*Given that, due to the current conditions, there can only be 18 students, it is recommended […] to send guides*” [Video 10/09/2021].

Secondly, the LPs were conditioned *by the Colombian curricular guidelines* (Colombia-MEN, 2016): the LPs cited curricular standards, and used examples and learning evidence suggested in those documents. As previously reported, the LPs followed structural guidelines, as follows: introduction and explanation [Doc: CP_1]; review of definitions and work based on two tasks [Doc: CP_3]; inquiry about previous knowledge, explanations, exercises, and tasks [Doc: CP_4]; theoretical review, learning-deepening activities, and review of contents [Doc: CP_5]. These decisions reflect PMTs’ desire to follow a series of conventions commonly used in schoolwork guides.

Thirdly, the *consideration of the LPs as evaluative items of the course* constituted a rule that regulated its design. LPs were required to include the use of technology in pandemic conditions, so PMTs had the task of recognizing such conditions and considering parameters and biosafety protocols, such as avoiding work groups and supporting virtual work and, eventually, face-to-face work once the conditions for returning to the classroom were met. In correspondence with these conditions, the PMTs expressed aspects such as the following: “*guaranteeing that the class session reaches all students*” [Doc: CP_3], “*we are still in a pandemic […] so it is recommended to use tools that are easy to access or master*” [Doc: CP_5], or “*the pandemic situation […] has led teachers to focus on pedagogical models that allow students to participate both virtually and face-to-face*” [Doc: CP_2].

#### Pandemic and community

The LPs were conditioned by both the educational legislation of the institutions and the community’s capacity of access to digital technological tools; therefore, the plans revealed the influence of the pandemic and the technology through the presented perceptions of the educational community. For example, for Ana, her work was intended to promote interactivity between teachers and students and encourage the use of technological resources; for Isabel, there were challenges for teachers beyond classroom work, including the possibility that students work from home, with their families and the available technologies; and for Alejandro, the pandemic generated changes that should be faced not only by educational control entities, but by all the educational actors. As we see it, the course generated interactions between the group of students and the teacher and was a discussion space about school processes in public educational institutions, promoting new points of view about the environment in which the school activities should be developed. The subjects (PMTs) and the educational community interacted from different scenarios with forms mediated by rules that were reorganized according to the pandemic and with tools that should guarantee access to the ‘new reality’ and to the environments in which mathematics is taught.

#### Pandemic: subjects and work distribution

The PMTs considered themselves as future actors in educational contexts; consequently, they designed LPs that brought together their vision about working in classrooms in pandemic conditions, the possible uses of technological devices and media, and state regulations on education. Since the PMTs were free to work in groups or develop their proposals independently, four of them decided to work individually and three of them grouped together to construct a single proposal [CP_1]. One limitation of this research is that the virtual nature of the course did not allow us to obtain evidence of interactions between the group members to identify how responsibilities were distributed during the planning and elaboration of their design. However, in the presentation that this group made to their classmates in the course, it was noticeable that each participant assumed a leading role in the elaboration of the work [video 27/08/2021] and developed tasks that contributed to consolidate the group. This, according to Russell (2009), gives shape to a co-construction; if the activity is collective, there emerges a collective subject that develops the activity (Lektorsky, 2009).

**Fig. 3:**
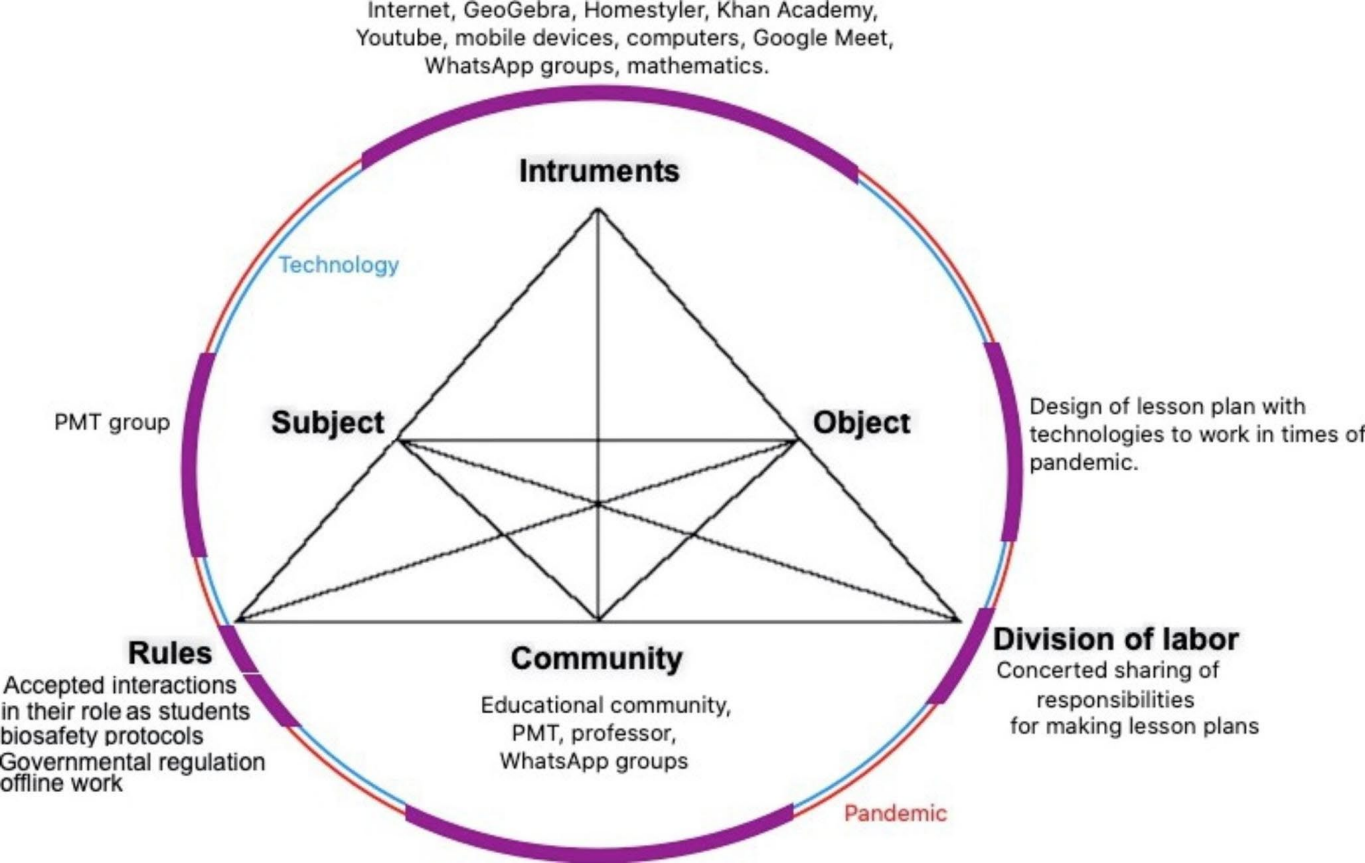
Agency of technology (blue), pandemic (red) and shared agency (purple) in the Activity System

The process of constructing a LPs offers evidence, as we have shown, that the pandemic and the available technology modified both the characteristics of the academic work and the students’ vision of what was happening in the classroom. Analyzing how to answer the questions suggested by Mwanza (2001), and what the characteristics and nuances in which the course was developed were, allowed us to identify the elements that were added to the nodes of the activity system (Fig. 3) and the incidence and impact that each one of them had in the whole system. As aforementioned, technology had specific roles in the LPs. These recreate similar uses in pre-pandemic times. However, the pandemic transformed classrooms and learning environments, so, technology took on other roles to ensure interaction, participation and communication amid social distancing and confinement. That is, technology-to-teaching in pandemic (Fig. 3. purple line).

## Discussion

In this study we investigated the roles that PMTs attributed to technologies for future teaching practices in the midst of pandemic conditions. The results show two different roles of technology in the LPs. On the one hand, *technology was used as a reorganizer of the ways of teaching in times of pandemic;* on the other hand, *technology was used to imitate pre-pandemic lectures*. Research has shown that teaching with technology can improve mathematics learning (Hillmayr et al., 2020). In our research, technology was used for mathematical work (for example, visualization, conceptual and procedural exploration) and as informational and collaborative tutoring systems; both uses existed in pre-pandemic mathematics education (Hillmayr et al., 2020).

In its role as reorganizer, technology focused on ‘alternation’ needs, that is, combining face to face and online teaching tasks such as providing online materials and enabling collaboration between students. For mathematical work, technologies supported both technical and conceptual mathematical activities; in the research literature, technical activities are mainly procedural execution tasks, while conceptual activities refer to inquiry, exploration, conjecture, and justification (Erens & Eichler, 2018). In their role as imitators, videoconferencing systems were proposed to reproduce lectures, tutoring systems were seen as repositories, and for mathematical work, technology was reduced to technical mathematical activities. These results supported pre-pandemic research that studied pragmatic, functional, experiential, critical and ethical needs of the professional development of teachers (Carmona-Mesa & Villa-Ochoa, 2017) and authors reported that for some teachers, technologies only fulfilled the basic function of an artifact for information checking and transmission (Souto & Borba, 2016).

The COVID-19 pandemic has reshaped research agendas and the way humans relate and engage in education (Borba, 2021). This means that the SARS-CoV-2 virus is an agent (thing) that has *conditional* agency, that is, it produces effects (Kaptelinin & Nardi, 2006). In this study, description of these effects on the components of the activity system are provided (Fig. 3). The pandemic has forced educational actors to generate and review ongoing actions to control the spread of the virus; learning environments must follow government regulations and institutional orientations and also respect individual particularities. The need to preserve health and life made schools and governments, as social entities, establish rules to limit physical contact and provide resources and guidance for teaching. In this sense, these entities also have agency (Need-based and Delegated agencies, Kaptelinin & Nardi 2006). In this study, the PMTs (subjects) have human agency, that is, the ability and the need to act (Kaptelinin & Nardi, 2006). In Tables 2 and 3, the purposes of these subjects, as declared in their LPs, are stated; although these purposes are consistent with potential actions for organization of classroom environments and mathematical work, it should also be noted that these LPs were built in response to a course completion task during alternation conditions at the time of the pandemic. This shows how the agency of these humans has a multi-conditional dimension. Finally, in this study, technologies are conceived as things that have agency; however, due to the nature of our data, we can only describe the agency that PMTs record in their LPs; that is, technologies (things) are agents in the sense that they act on behalf of another person (PMT) (Kaptelinin & Nardi, 2006). As shown in Tables 2 and 3, PMTs assigned functions to technologies for the reorganization of classroom environments, of mathematical work in the classroom, and sometimes to recreate activities of conventional pre-pandemic teaching. This shows differences between technology agency as assigned by PMTs and its potential as documented for research in pre-pandemic teaching (Villa-Ochoa & Suárez-Téllez, 2021; Hillmayr et al., 2020; Erens & Eichler, 2018) and during the pandemic (Alabdulaziz, 2021; Naidoo, 2020). Similarly to results reported by Alabdulaziz (2021) and Naidoo (2020), in our study, the PMTs recognized that the pandemic created conditions for technology to remain in the educational process permanently; however, unlike these studies, our results reinforce the need to transcend a simple positive perception and to recognize the agency of technology and how it could be integrated into curricula and school context conditions.

Although our research involved PMTs in a very specific Colombian context, it offers information to understand how an emergency can affect the design of a class and, therefore, considering the aforementioned literature, it also alerts us to possible changes in teacher education. In this sense, this study provides evidence in answer to the questions formulated by Engelbrecht et al., (2020), on the impact of COVID-19 on mathematics education; it was observed that the presence of the new virus forced immediate social actions to control its spread and, in turn, provoked reactions of the subjects according to the perceived risks.

Based on the data analysis, these risks are interpreted as contradictions that promoted actions in the PMTs. Consistent with the findings of Molina-Toro et al., (2022), technology, when introduced to the activity system, generates contradictions that are a source of learning. In this study, the perceived risk generated opportunities for the PMTs to become familiar with mathematics teaching under emergency conditions. In the study, it was observed that LPs were mainly organized to support the teaching of content, and to enable connectivity and communication between students and teachers. The analysis of the impact of the pandemic in the components of the activity system suggests that this method of organization is PMTs’ response to observed risks in the teaching activity, considering their perceptions of the possibilities and limitations of the technologies, and their interpretations of governmental and institutional norms and orientations. Specifically, PMTs perceived that students could not attend classrooms in a conventional way; the recognition of this fact explains why their main efforts to use technologies were centered in connectivity and access to information. On the other hand, PMTs knew cases in which in-service teachers reduced their teaching activity to the elaboration of written and evaluative materials; this fact helps to explain why there was a special emphasis on the organization of sequences of activities to promote conceptual and procedural understanding of contents. There is thus a correspondence between the risks perceived by the PMTs and their proposed LPs. This shows that technologies were perceived as a means to ‘overcome’ the limitations of these risks and achieve the proposed objectives, which represented a negotiation between ‘a certain teaching tradition’ and new demands due to the pandemic (i.e., rules in the activity system). Although some of the LPs demonstrated only basic uses of technology, the potential of its inclusion in future teaching activities will allow educational actors to gradually become familiar with it; furthermore, it is expected that, as PMTs get new technological experiences, they will recognize other risks and will refine their goals, generating more robust uses of technology and more adequate response actions.

Research literature informs on “agentic power” of digital technologies in mathematical thinking (Villa-Ochoa & Suárez-Téllez, 2021; Hillmayr et al., 2020) and by the Internet and platforms for the design of learning environments (Borba, 2012; Rosa & Orey, 2019; Agostinho & Reis, 2021), and presents interactive environments that promote participation, interaction, and knowledge production (Parra-Zapata et al., 2018). Despite this power, the results of this study raise concerns about how to integrate these research results adequately in the near future and in the midst of pandemic conditions. This study also highlights the need for researchers and teacher educators to continue making efforts to provide well-established conceptualizations about how mathematics education should be adapted for emergencies, and to bring together research and concrete actions to face such a contingency (Parra-Zapata & Villa- Ochoa, 2022).

## Conclusion

In this study we investigated the roles that PMTs attribute to technologies in the activity of planning mathematics lessons during a pandemic. Evidence shows that this activity system involved the use of an articulated set of Internet-based technologies as a response to perceived needs of connectivity and mathematical content teaching. The PMTs resorted to material devices (computers, tablets, and mobile phones) which, through their connection to the Internet, were integrated with videoconferencing platforms, social networks and instant messaging to access information and promote participation, communication, and interaction between subjects. The use of disciplinary software and applications (GeoGebra, web resources) was also integrated as a means to explore and visualize topics dynamically.

To investigate the roles that PMTs attribute to technologies, we used the notion of *agency* as a way to transcend the mere verification of the presence of a technology and its functionalities. We recognized PMTs as human agents and described the agency they delegated to technologies; we observed that these agencies were interweaved with the conditions of the pandemic and with the context of the course in which the LPs were designed. We recognized divergences between the agency that PMTs delegated to technologies and the agentic power that research literature has documented about technologies. We observed that the pandemic is a conditioning agent that imposes conditions on education; consequently, we described how the H-w-M collective identified its effects and planned actions to confront them. These results offer insights into how agencies are present in the activity system, and how H-w-M can be conceived as a collective with shared agency. These facts provide further evidence for future theorization of these notions in the H-w-M theoretical constructs, in order to refine and develop new analytical tools for research.

While these results reflect the proposals of PMTs in a specific preparation context, they support an important recommendation for teacher education programs worldwide. Considering the interrelations between agencies in H-w-M systems during the pandemic, there is a need to continue paying explicit attention to connections between technology, mathematics teaching/learning, and emergency conditions in order to promote actions that address teaching processes during general emergency situations. The COVID-19 pandemic generated a variety of crises in individuals and educational systems. More research is still needed to describe and understand these crises, contexts, and subject systems; but research must also promote immediate response actions in the face of crises generated by the pandemic or by future emergencies. Some important related topics include the concept of the home as a classroom, people’s emotions, limited Internet access and connectivity, and many others. In particular, we highlight the need to continue supporting PMTs in the creation and selection of tasks that, in addition to conceptual aspects, promote diverse learning experiences and adequate planning to respond to emergency conditions.

Although this study offers insights into how the pandemic affects educational planning with technology, it also suggests the need to investigate whether these results are specific to a geographic region or if they are present in other regions. The conclusions of this study show how this group of future teachers conceives of mathematics teaching activities with technology. While existing research has shown that the pandemic generated favorable conditions for technology to be integrated into the daily life of schools, this study suggests the need to go further and analyze these conditions, the opportunities they offer, the roles and types of technologies available, and their ways of articulation with mathematical knowledge, considering also the environmental and cultural conditions required for student to learn during an emergency. Such research would promote early preparation actions and would avoid simply perpetuating existing pre-pandemic curricular guidelines.

Although the LPs offer important information about how the PMTs see their future teaching practice, it is also true that they are limited, since we cannot observe how the LPs were implemented and adjusted in real school practices. Therefore, it is recommended that future research study how the agencies interact with the ‘resistance’ offered by real school conditions; this could potentially help reduce the differences between the agency assigned by PMTs to technology and the agency informed by previous research. Finally, throughout this study, we reported the presence of Internet-based technologies that had a delegated agency for communication, access to information, participation, and collaboration; additionally, we reported the presence of technologies for mathematical work to which an agency for conceptual and procedural mathematics was assigned. In a certain way, these agencies were observed to be disjointed. Future research could provide insight on other (new) technologies with joint agentic power for collaborative mathematics.
